# Impaired Kv4.2-DPP6 complex dynamics in a mouse model of Angelman syndrome

**DOI:** 10.3389/fncel.2026.1953686

**Published:** 2026-10-21

**Authors:** Jia-Hua Hu, Ying Liu, Cole Malloy, Dax A. Hoffman

**Affiliations:** Molecular Neurophysiology and Biophysics Section, The Eunice Kennedy Shriver National Institute of Child Health and Human Development, Bethesda, MD, United States

**Keywords:** Angelman syndrome (AS), dendritic excitability, DPP6, Kv4.2 (KCND2), membrane trafficking, UBE3A

## Abstract

**Introduction:**

Angelman syndrome (AS) is a neurodevelopmental disorder caused by loss of neuronal expression of the E3 ubiquitin ligase UBE3A and is associated with altered neuronal excitability. We previously identified the dendritic A-type K+ channel subunit Kv4.2 as an activity-dependent substrate of UBE3A. However, where UBE3A interacts with Kv4.2 during activity-dependent trafficking and how this pathway regulates Kv4.2 channel complexes and dendritic distribution remain unclear..

**Methods:**

We combined live-cell bungarotoxin labeling and biochemical surface-labeling approaches to examine UBE3A association with surface-derived Kv4.2. Co-immunoprecipitation and immunoblotting were used to assess Kv4.2 association with the auxiliary subunit DPP6 and activity-dependent Kv4.2 phosphorylation in wild-type (WT), UBE3A-deficient (AS), and DPP6 knockout (KO) mice. Kv4.2 immunostaining and quantitative line-scan analysis were used to examine its proximal-to-distal distribution in hippocampal CA1 dendrites.

**Results:**

UBE3A preferentially associated with internalized rather than surface-resident Kv4.2, supporting a post-endocytic site of UBE3A action. In AS mice, Kv4.2–DPP6 association was reduced and failed to undergo further activity-dependent remodeling following pentylenetetrazole (PTZ)-induced seizures, despite unchanged DPP6 expression and preserved basal Kv4.2 phosphorylation. In contrast, DPP6 deficiency increased basal Kv4.2 phosphorylation at T602 and T607 and disrupted its activity-dependent phosphorylation dynamics, indicating reciprocal regulation between Kv4.2 phosphorylation and DPP6 association. DPP6KO mice exhibited impaired Kv4.2 gradients along both apical and basal CA1 dendrites. Similarly, AS mice showed impaired dendritic Kv4.2 gradients and reduced activity-dependent redistribution following kainic acid-induced seizures.

**Conclusion:**

These findings support a model in which neuronal activity promotes remodeling and internalization of Kv4.2-containing channel complexes, followed by UBE3A-dependent processing of the post-endocytic Kv4.2 pool. Loss of UBE3A disrupts Kv4.2–DPP6 complex dynamics and dendritic Kv4.2 organization, thereby reducing the capacity for activity-dependent channel redistribution. These results identify UBE3A as an important regulator of Kv4.2 homeostasis and provide a mechanistic link between UBE3A deficiency, altered dendritic ion-channel organization, and neuronal dysfunction in Angelman syndrome.

## Graphical abstract

**Figure:**
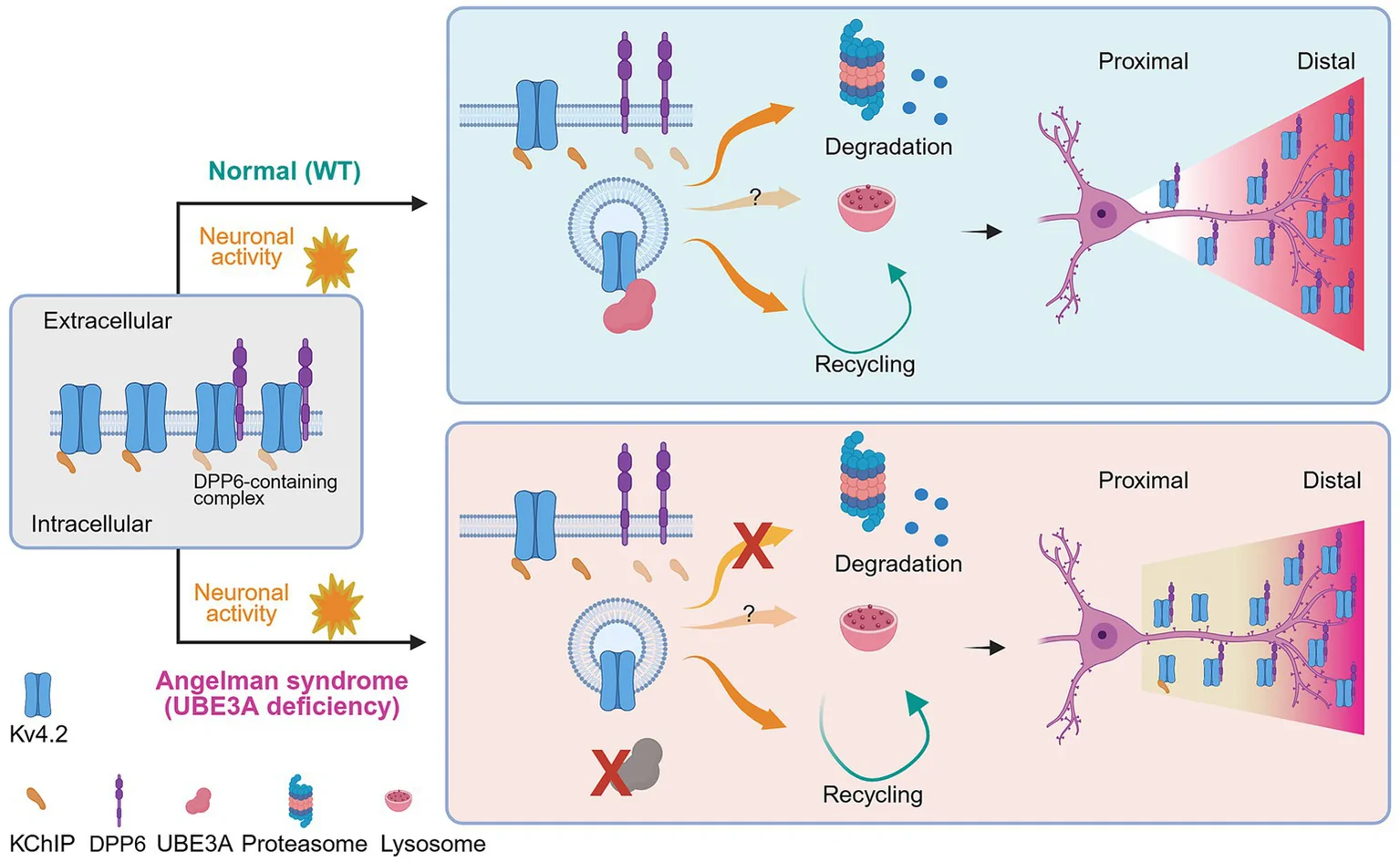
Proposed model illustrating how UBE3A regulates the turnover and distribution of internalized Kv4.2 to maintain proper dendritic excitability. Neuronal activity induces the dissociation of Kv4.2 from DPP6, followed by Kv4.2 internalization. Under normal conditions (WT), UBE3A preferentially binds to internalized Kv4.2 and promotes its subsequent processing and turnover. Internalized Kv4.2 may undergo proteasomal degradation, lysosomal degradation, and/or recycling, although the relative contributions of these pathways remain to be determined. This process maintains the dendritic gradient of Kv4.2 expression in hippocampal CA1 neurons and preserves normal dendritic excitability. In Angelman syndrome (AS), where UBE3A is absent, activity-dependent internalized Kv4.2 is no longer efficiently targeted for proteasomal degradation and may instead undergo increased recycling back to the plasma membrane. As a result, the normal dendritic gradient of Kv4.2 is disrupted, leading to impaired dendritic excitability in hippocampal CA1 neurons. Created with BioRender.com.

## 1 Introduction

Angelman syndrome (AS) is a severe neurodevelopmental disorder caused by the loss of functional expression of the maternally inherited UBE3A gene in neurons (Buxton et al., 1994; Magenis et al., 1987; Nelen et al., 1994; Kishino et al., 1997). Due to genomic imprinting, the paternal UBE3A allele is epigenetically silenced in most regions of the brain, rendering neuronal UBE3A expression largely dependent on the maternal allele (Albrecht et al., 1997). Consequently, maternal deletions or mutations of UBE3A, paternal uniparental disomy, or imprinting defects all result in a loss of UBE3A function in neurons (Buiting et al., 2016). Clinically, Angelman syndrome is characterized by intellectual disability, severe speech impairment, motor dysfunction, epilepsy, and a distinct behavioral phenotype that includes heightened sociability and frequent laughter (Mertz et al., 2013; Elgersma and Sonzogni, 2021). At the cellular and circuit levels, loss of UBE3A leads to widespread synaptic dysfunction, including disruptions in excitatory–inhibitory balance, impaired long-term potentiation, and abnormal neuronal excitability (Sell et al., 2021; Rotaru et al., 2018; Avagliano Trezza et al., 2019). These deficits are thought to underlie the neurological and behavioral abnormalities observed in patients. At present, no FDA-approved disease-modifying therapy exists for AS, and clinical management remains entirely symptomatic and supportive (Samanta, 2026).

The UBE3A gene encodes the ubiquitin protein ligase E3A, also known as E6-AP, which primarily acts as an E3-ligase in ubiquitin-dependent protein degradation by the 26S proteasome (Matentzoglu and Scheffner, 2008; Krzeski et al., 2024). Deficits of UBE3A lead to accumulation of its target proteins and thus dysregulate neuronal function. To elucidate the molecular and circuit mechanisms associated with UBE3A deficiency, a variety of genetically engineered mouse models have been developed that recapitulate key features of the disorder. Among these, the maternal UBE3A knockout mouse (*Ube3a^m−/p+^*) is the most widely used model, in which the maternal allele is selectively disrupted to mimic the neuronal loss of UBE3A observed in Angelman syndrome (Heck et al., 2008; Jiang et al., 1998; van Woerden et al., 2007). These mice exhibit hallmark phenotypes, including motor impairments, deficits in learning and memory, increased seizure susceptibility, and altered synaptic plasticity (Rotaru et al., 2018; Heck et al., 2008; Jiang et al., 1998; Born et al., 2017). Electrophysiological studies have demonstrated impairments in long-term potentiation and changes in neuronal excitability across multiple brain regions, including the hippocampus and cortex (Jiang et al., 1998; Wallace et al., 2012). In addition, structural and functional abnormalities in dendritic spines and synaptic transmission further support a critical role for UBE3A in maintaining synaptic integrity (Sell et al., 2021; Hu et al., 2025).

Angelman syndrome mouse models have enabled the identification of molecular substrates and signaling pathways regulated by UBE3A, including ion channels, synaptic scaffolding proteins, and activity-dependent regulators of plasticity (Krzeski et al., 2024; Margolis et al., 2010; Sun et al., 2015; Sun et al., 2019). In addition, we previously identified Kv4.2 as a neuronal activity-dependent substrate of UBE3A (Hu et al., 2025). The activity-dependent association of Kv4.2 and UBE3A is unique and plays a critical role in regulating DPP6-containing Kv4.2 protein complexes.

Kv4.2, a member of the Shal-related Kv channel family, is the pore-forming subunit expressed in the dendrites of hippocampal CA1 pyramidal neurons (Hoffman et al., 1997). Kv4.2 is localized to the somatodendritic compartment, where it controls subthreshold excitability and dendritic signal integration (Hoffman et al., 1997). Kv4.2 channels function as part of macromolecular protein complexes that include auxiliary subunits such as K^+^ channel interacting proteins (KChIP1–4) and dipeptidyl peptidase-like proteins DPP6 and DPP10 (Pongs and Schwarz, 2010; Kise et al., 2021). These auxiliary proteins are essential for regulating Kv4.2 channel trafficking, surface expression, and gating properties (Pongs and Schwarz, 2010). In particular, DPP6 is a type II transmembrane protein that increases Kv4.2 membrane expression, single channel conductance and accelerates the inactivation and recovery from inactivation of Kv4 subunit-containing channels (Nadal et al., 2001; Kaulin et al., 2009). In hippocampal CA1 pyramidal neurons, Kv4.2-mediated currents exhibit a gradient that increases from the soma to distal dendrites (Hoffman et al., 1997), a property that is abolished in DPP6 knockout mice, indicating a critical role for DPP6 in establishing dendritic channel distribution (Sun et al., 2011). In addition to its Kv4.2-dependent functions, DPP6 has also been implicated in synaptic development independently of Kv4.2 (Lin et al., 2013), and genetic studies have linked DPP6 and DPP10 to neurodevelopmental and neurodegenerative disorders (Lin et al., 2020; Lin L. et al., 2022; Li et al., 2024), suggesting broader functional significance of this complex.

Our previous studies revealed a p38-Pin1-dependent mechanism of Kv4.2 regulation by UBE3A in mouse brain (Hu et al., 2025; Hu et al., 2020). Kv4.2 is phosphorylated by the proline-directed kinase p38 at threonine 607 (T607) in response to neuronal activity. This modification promotes binding of the prolyl isomerase Pin1, which catalyzes conformational changes that disrupt Kv4.2 interaction with DPP6 and binding to UBE3A for its activity-dependent degradation (Hu et al., 2025; Hu et al., 2020). Despite substantial progress, the process that underlies UBE3A binding to Kv4.2 and how it alters Kv4.2 distribution is still unclear. In this study, we investigate where UBE3A binds to Kv4.2 and how this pathway regulates Kv4.2 distribution in mouse hippocampus in AS mouse model.

## 2 Results

### 2.1 UBE3A preferentially associates with internalized Kv4.2

We previously identified Kv4.2 as a neuronal activity-dependent substrate of UBE3A (Hu et al., 2025), suggesting that the interaction between these proteins is dynamically regulated rather than constitutive. Neuronal activity promotes phosphorylation of Kv4.2 at T607, leading to dissociation from the auxiliary subunit DPP6 and subsequent channel internalization (Hu et al., 2020). Based on this model, we hypothesized that UBE3A preferentially interacts with internalized, rather than surface-resident, Kv4.2 channels.

To directly test this, we utilized a bungarotoxin-binding site-tagged Kv4.2 construct (BBS-Kv4.2), which enables selective live labeling of surface-expressed channels. HEK293T cells were co-transfected with UBE3A and BBS-Kv4.2, and surface Kv4.2 was labeled with rhodamine-conjugated α-bungarotoxin (Rh-BTX). Following surface labeling, cells were maintained either at 4 °C to restrict membrane trafficking or transferred to 37 °C to permit internalization and intracellular trafficking of the labeled Kv4.2 population. Thus, the Rh-BTX signal could be used to follow the fate of Kv4.2 that was initially present at the cell surface. Under conditions that inhibit membrane trafficking (4 °C), surface-labeled Kv4.2 exhibited minimal colocalization with intracellular UBE3A (Figure 1A), indicating limited interaction at the plasma membrane. In contrast, incubation at 37 °C for 45 min to permit endocytosis and intracellular trafficking resulted in a marked increase in Kv4.2–UBE3A colocalization (Figure 1A). Because the Kv4.2 population was labeled before the temperature shift, the increased colocalization at 37 °C indicates that UBE3A preferentially associates with Kv4.2 after the surface-labeled channel undergoes intracellular trafficking.

**Figure 1:**
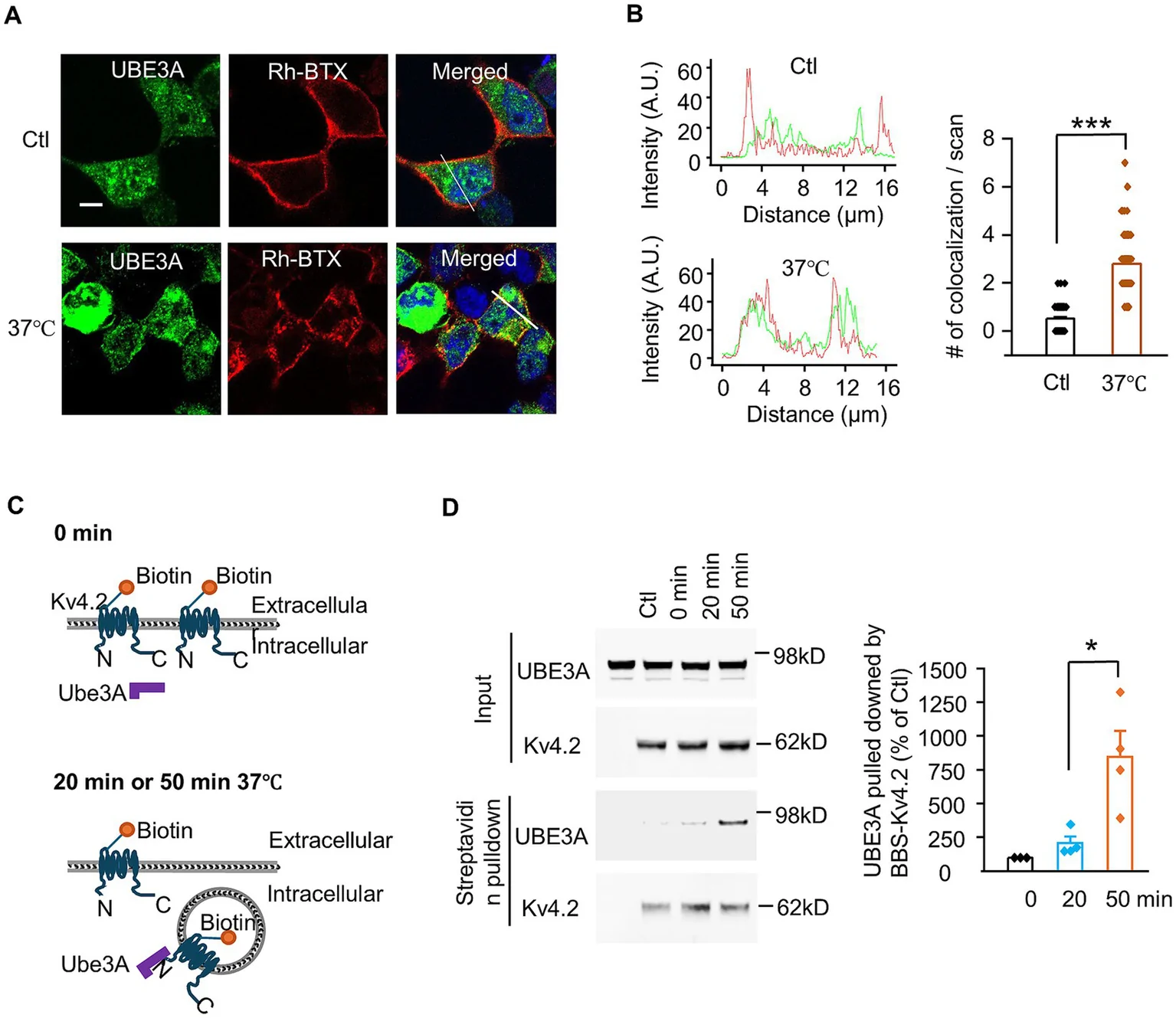
UBE3A binds to internalized Kv4.2 rather than surface Kv4.2. **(A)** UBE3A colocalization with Kv4.2 is increased when permitting Kv4.2 internalization (37 °C, 45 min) compared to control (4 °C, 45 min) when expressed in HEK293T cells. Scale bars: 5 μm. **(B)** Colocalization was quantified using line scan in Image J. Data was obtained from 21 cells, and 4 scans of each cell for each group. Two-sample *t*-test, ***p < 0.001. **(C)** Diagram of surface labeled Kv4.2 pulling down UBE3A when expressed in HEK293T cells. **(D)** Kv4.2 pulls down more UBE3A when permitting Kv4.2 internalization (37 °C, 20 or 50 min) than control (4 °C, 50 min) in HEK293T cells. *n =* 3, 4, 4 for Ctl, 20 min and 50 min, respectively. Two-sample *t*-test, *p < 0.05, 50 min vs. 20 min.

To further validate these observations biochemically, we labeled surface Kv4.2 with biotinylated BTX, isolated labeled channels using streptavidin pull-down, and assessed UBE3A association by immunoblotting. Consistent with imaging results, Kv4.2–UBE3A interaction was barely detectable under restricted trafficking conditions but was robustly increased following incubation at 37 °C (Figure 1B). The association increased with the duration of incubation at 37 °C, with greater UBE3A recovery observed at 50 min than at 20 min. Together, these findings demonstrate that UBE3A preferentially binds to internalized Kv4.2 rather than surface-resident Kv4.2, supporting a model in which activity-dependent channel endocytosis facilitates UBE3A-mediated regulation.

### 2.2 Kv4.2–DPP6 association is reduced in AS mice

Given that UBE3A regulates Kv4.2 stability and that DPP6 is a key auxiliary subunit required for Kv4.2 trafficking and function, we next examined whether UBE3A deficiency alters DPP6 expression and its association with Kv4.2. As previously reported, Kv4.2 protein levels are elevated in UBE3A-deficient mice (designated AS mice), an animal model of AS, reflecting impaired UBE3A-mediated degradation (Hu et al., 2025). However, biochemical fractionation of hippocampal tissue revealed that DPP6 is predominantly localized to non-postsynaptic density (non-PSD) compartments and that its overall abundance is unchanged across synaptosomal, PSD, and non-PSD fractions in AS mice compared to wild-type controls (Figure 2A). Thus, loss of UBE3A does not appear to produce a major change in either the total abundance or biochemical fractionation profile of DPP6. We next asked whether UBE3A directly associates with DPP6. Co-immunoprecipitation experiments in HEK293T cells expressing DPP6 and UBE3A showed no detectable interaction between UBE3A and DPP6 (Figure 2B). These results suggest that DPP6 is unlikely to be directly regulated through stable binding to UBE3A.

**Figure 2:**
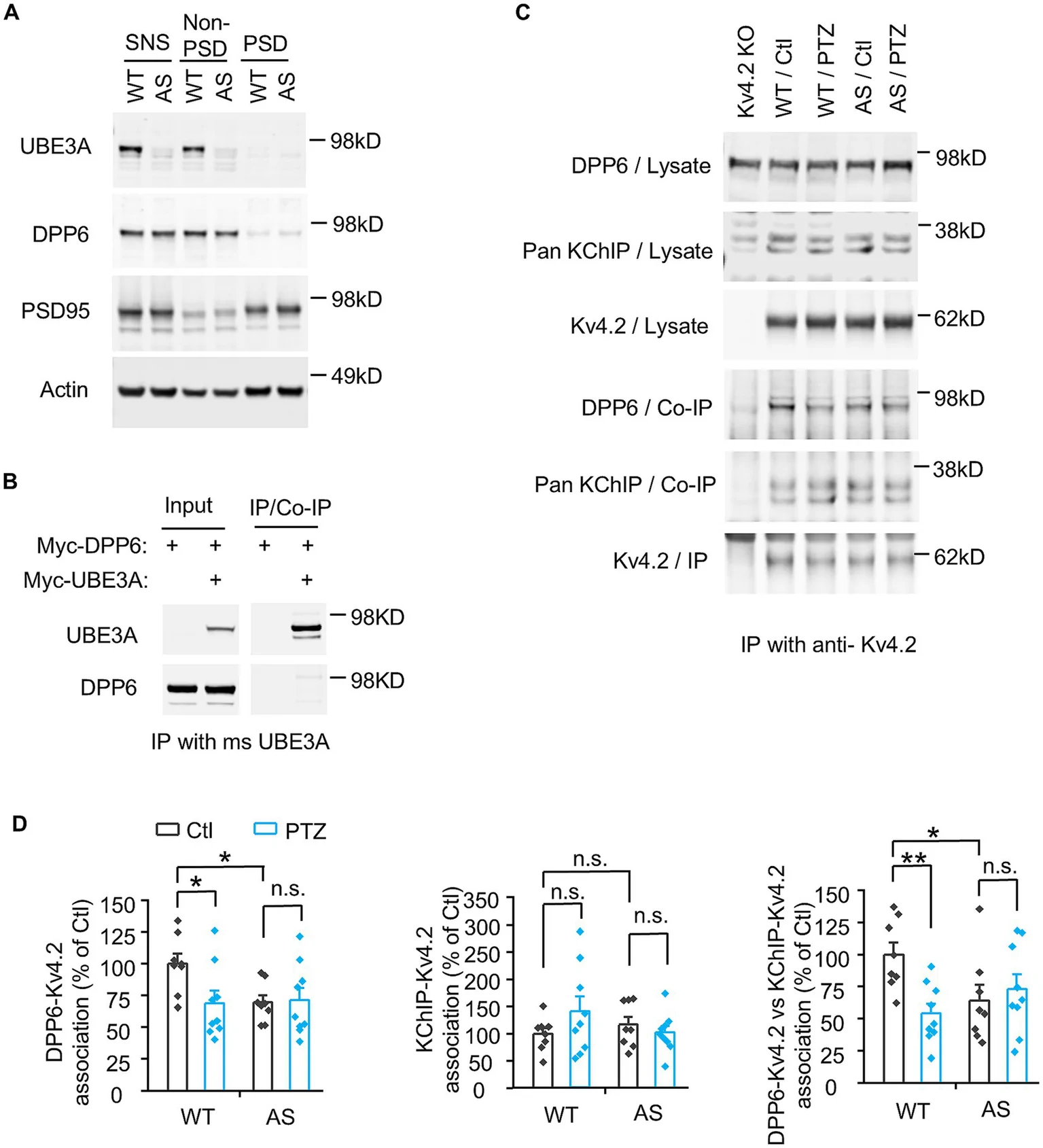
DPP6-Kv4.2 association is reduced in AS mice. **(A)** DPP6 protein level is normal in crude synaptoneurosomal fractions (SNS), non-PSD fractions and PSD fractions in AS mouse hippocampus. **(B)** DPP6 does not co-IP with UBE3A when expressed in HEK293T cells. **(C)** Co-immunoprecipitation analysis showed that DPP6-Kv4.2 association is reduced in AS mice and WT mice with PTZ administration (60 mg/kg, i.p. 30 min), while KChIP-Kv4.2 association is not significantly altered. **(D)** Statistic analysis of **(C)**. *n =* 8, 9, 8, 9 for WT/Ctl, WT/PTZ, AS/Ctl, AS/PTZ, respectively. Two-way ANOVA with Fisher’s LSD test. There is no interaction between genotype and treatment for DPP6-Kv4.2 association or KChIP-Kv4.2 association, but interaction for DPP6-Kv4.2 vs. KChIP-Kv4.2 association, *F*(1,30) = 7.048, *p* = 0.0126. *p < 0.05, **p < 0.01.

We next examined if Kv4.2-DPP6 association is impaired in AS mice. Kv4.2 was immunoprecipitated from brain tissue, and associated DPP6 and KChIP proteins were measured by immunoblotting. KChIPs were included as another major class of Kv4 auxiliary proteins, allowing us to determine whether loss of UBE3A broadly disrupts Kv4.2 auxiliary-subunit interactions or preferentially affects its association with DPP6. Co-immunoprecipitation assays revealed a significant reduction in Kv4.2–DPP6 association in AS mice, whereas the interaction between Kv4.2 and KChIP auxiliary subunits remained unaffected (Figures 2C,D). As previously reported (Hu et al., 2020), seizure induced by intraperitoneal (IP) injection of pentylenetetrazole (PTZ) decreased Kv4.2-DPP6 association. We then examined if PTZ administration alters Kv4.2-DPP6 association in AS mice and their WT littermates. Co-immunoprecipitation experiments showed that Kv4.2-DPP6 association but not Kv4.2-KChIP association was significantly reduced in WT mice administered PTZ (60 mg/kg, 30 min) compared to control mice (Figures 2C,D). However, the PTZ-mediated reduction of Kv4.2-DPP6 association is abolished in AS mice (Figures 2C,D). These data suggest that UBE3A is required for activity-dependent remodeling of Kv4.2–DPP6 interactions.

### 2.3 Kv4.2 phosphorylation dynamics are preserved in AS mice but disrupted in DPP6-deficient mice

Our previous work identified phosphorylation-dependent regulation as an important upstream step controlling Kv4.2–DPP6 association. In particular, activity-dependent phosphorylation of Kv4.2 at T607 promotes Pin1 binding and isomerization, which facilitates remodeling of the Kv4.2 complex and dissociation from DPP6 (Hu et al., 2020). The reduced basal Kv4.2–DPP6 association and altered activity-dependent remodeling observed in AS mice therefore raised the possibility that Kv4.2 phosphorylation might itself be abnormal in the absence of UBE3A. To test this possibility, hippocampi from AS mice and their WT littermate controls mice were isolated and Kv4.2 proteins were enriched with Kv4.2 antibodies. Samples were then subjected to western blot assay for Kv4.2 phosphorylation using pT602 and pT607 antibodies. The result showed that T602 and T607 phosphorylation of Kv4.2 are normal in AS mice compared to WT littermate controls (Figure 3A), indicating that steady-state phosphorylation is not affected by UBE3A loss. We next assessed activity-dependent phosphorylation following PTZ-induced seizures. Consistent with prior studies, PTZ increased T607 phosphorylation in wild-type mice without affecting T602. In AS mice, PTZ similarly increased T607 phosphorylation but also induced a significant increase in T602 phosphorylation, indicating altered activity-dependent phosphorylation dynamics (Figure 3A). These findings suggest that UBE3A is not required for basal phosphorylation of Kv4.2 but may alter events downstream of activity-dependent phosphorylation and channel-complex remodeling.

**Figure 3:**
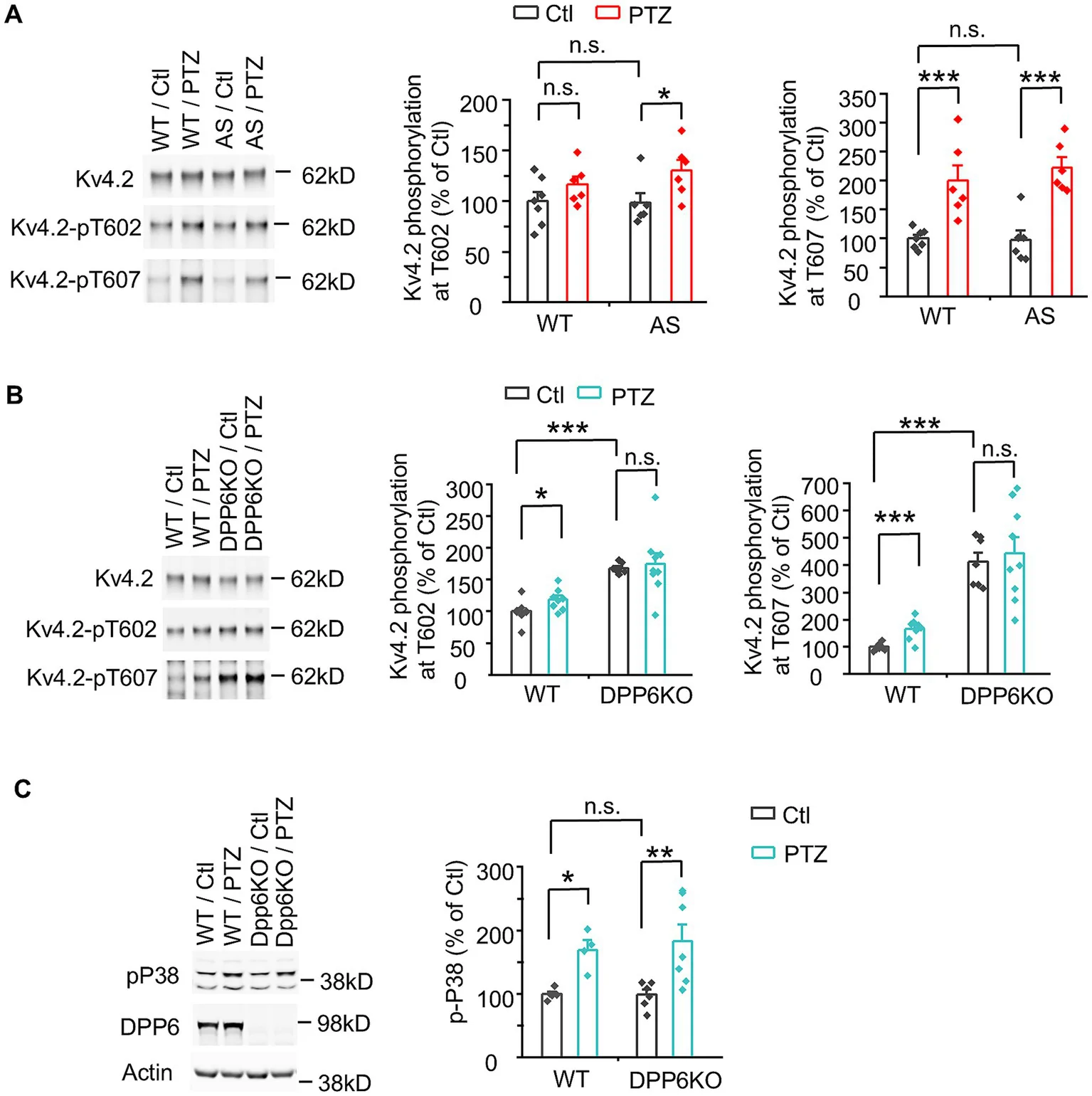
Altered Kv4.2 phosphorylation at Pin1 sites in AS and DPP6KO mice. **(A)** Kv4.2 phosphorylation at Pin1 sites in AS mice. *n =* 7, 6, 6, 6 in WT/Ctl, WT/PTZ, AS/Ctl, AS/PTZ, respectively. Two-way ANOVA with Fisher’s LSD test. Main effect of AS: *F*(1, 21) = 0.4312, *p* = 0.5185 (T602 phosphorylation); *F*(1, 21) = 0.3374, *p* = 0.5675 (T607 phosphorylation). *p < 0.05, ***p < 0.001. **(B)** Kv4.2 phosphorylation at Pin1 sites in DPP6KO mice. *n =* 8, 8, 7, 9 in WT/Ctl, WT/PTZ, DPP6KO/Ctl, DPP6KO/PTZ, respectively. Two-way ANOVA with Fisher’s LSD test. Main effect of DPP6KO: *F*(1, 28) = 35.36, *p* < 0.0001 (T602 phosphorylation); *F*(1, 28) = 65.04, *p* < 0.0001 (T607 phosphorylation). Two-sample t-test, *p < 0.05, ***p < 0.001. **(C)** p38 phosphorylation in DPP6KO mice. *n =* 5, 4, 6, 7 in WT/Ctl, WT/PTZ, DPP6KO/Ctl, DPP6KO/PTZ, respectively. Two-way ANOVA with Fisher’s LSD test. Main effect of DPP6KO: *F*(1, 18) = 0.1274, *p* = 0.1274. *p < 0.05, **p < 0.01.

In contrast, analysis of DPP6 knockout (DPP6KO) mice revealed a distinct phenotype. Basal phosphorylation at both T602 and T607 was significantly elevated in DPP6KO mice relative to controls (Figure 3B). This is consistent with our previous finding that DPP6 expression significantly decreases Kv4.2 phosphorylation at T602 and T607 when co-expressed in HEK293T cells (Hu et al., 2025). Moreover, PTZ-induced increases in Kv4.2 phosphorylation were absent in DPP6KO mice, indicating a loss of activity-dependent phosphorylation dynamics. Notably, phosphorylation of p38 MAPK, the kinase responsible for T602 and T607 phosphorylation, was unchanged at baseline and following PTZ treatment (Figure 3C), suggesting that upstream kinase activation is intact. These results indicate that DPP6 is required for proper regulation of Kv4.2 phosphorylation dynamics, potentially by organizing signaling-competent channel complexes.

### 2.4 Kv4.2 gradient is impaired in DPP6KO mice

A-type K^+^ currents increase in density from the soma to distal dendrites in CA1 hippocampal pyramidal neurons (Hoffman et al., 1997), suggesting a gradient distribution of Kv4.2 protein along the apical dendrites of CA1 pyramidal neurons. We also reported previously that DPP6 establishes the A-type K^+^ gradient along the apical dendrites of CA1 pyramidal neurons (Sun et al., 2011). However, whether loss of DPP6 also disrupts the underlying spatial distribution of Kv4.2 protein along CA1 dendrites had not been directly examined. We therefore used Kv4.2 immunostaining to determine whether the electrophysiologically defined DPP6-dependent current gradient is accompanied by a corresponding gradient in Kv4.2 protein abundance.

Immunostaining experiments confirmed that Kv4.2 protein is distributed in a gradient along both apical dendrites (quantified proximal about 10 μm away from *stratum pyramidale* (SP) and distal 130 μm away from SP for *stratum radiatum* (SR)) and basal dendrites [quantified proximal about 10 μm away from SP and distal 70 μm away from SP for *stratum oriens* (SO)] in WT mouse hippocampus (Figures 4A,B). We reported previously that the Kv4.2 gradient along CA1 apical dendrites was impaired using electrophysiological recordings in DPP6KO mice (Sun et al., 2011). Immunostaining was performed in DPP6KO mice, and the results showed that the Kv4.2 gradient was significantly impaired on both apical and basal dendrites (Figures 4A,B).

**Figure 4:**
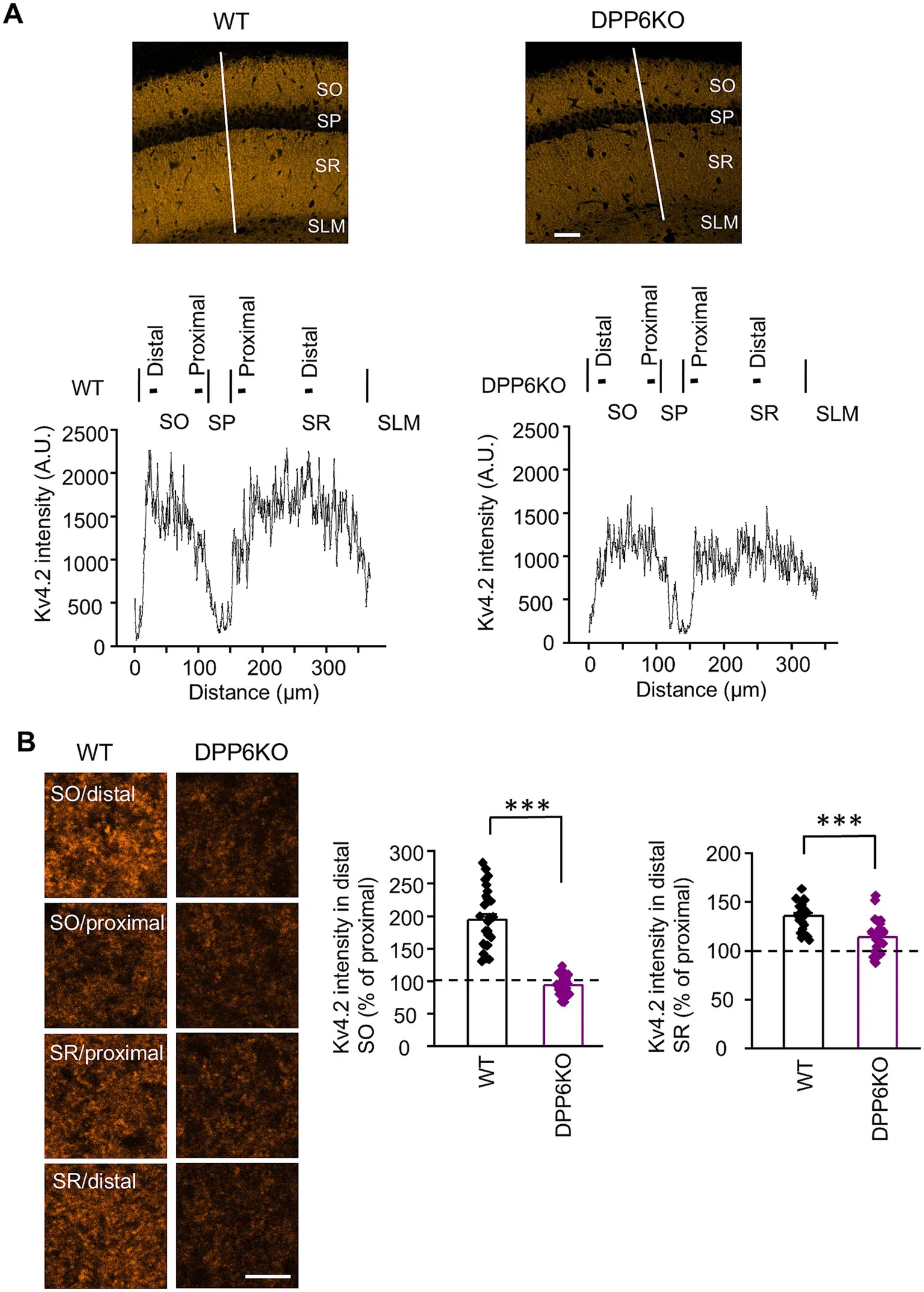
Gradient distribution of Kv4.2 along dendrites in CA1 is impaired in DPP6KO mice. **(A)** Immunostaining analysis showed that Kv4.2 is expressed in basal dendrites and apical dendrites as a gradient in CA1 in WT hippocampus. The gradient expression of Kv4.2 is impaired in the same area of DPP6KO mice. Line scan showed the representative Kv4.2 signal in each slice. Scale bars: 50 μm. SO: stratum oriens, SP: stratum pyramidale, SR: stratum radiatum, SLM: stratum lacunosum moleculare. **(B)** Representative images of distal/proximal SO and distal/proximal SR, and statistical analysis of Kv4.2 gradient showed in **(A)**. Data presented as Kv4.2 level in distal dendrites normalized to proximal dendrites. *n =* 3 mice for each group. Three slides were used for each mouse and three-line scans for each slide. Two-sample *t*-test, ***p < 0.001, Scale bars: 5 μm.

### 2.5 Kv4.2 gradient is disrupted in AS mice

We hypothesized that Kv4.2 distribution on dendrites is impaired given disrupted Kv4.2-DPP6 association in AS mice (Figures 2C,D). We examined the Kv4.2 gradient in AS mice using the same approach as described above. The Kv4.2 gradient was significantly impaired along both basal and apical dendrites in AS mice compared to WT control mice (Figures 5A,B). In addition, we examined if kainic acid (KA)-induced seizure altered the Kv4.2 protein distribution as it reduced Kv4.2 protein level (Hu et al., 2025). We found that the distribution of the Kv4.2 protein gradient along both apical and basal dendrites is affected when WT mice were administered with KA compared to control (Figures 5A,B), suggesting neuronal activation is associated not only with biochemical remodeling of Kv4.2-containing complexes but also with redistribution of Kv4.2 across CA1 dendritic compartments. AS mice administered with KA showed a loss of the Kv4.2 gradient in SO compared to AS control mice as well, but the degree of Kv4.2 gradient impairment by KA in AS mice was significantly less compared to that in WT mice (Figures 5A,B), suggesting KA has less impact of Kv4.2 gradient loss on AS mice than on WT littermates.

**Figure 5:**
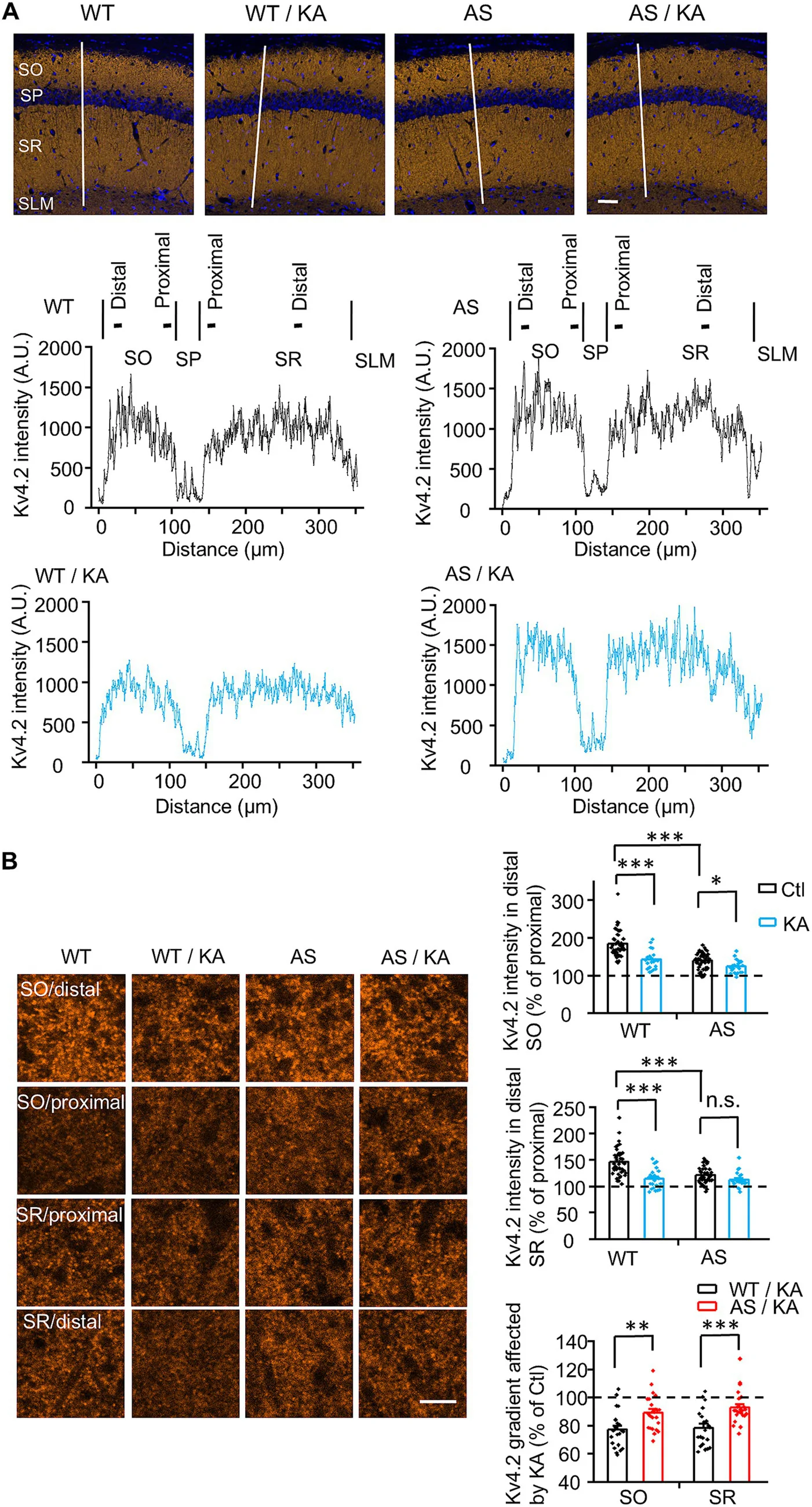
Gradient distribution of Kv4.2 along dendrites in CA1 is impaired in AS mice. **(A)** Immunostaining analysis showed that Kv4.2 is expressed as a gradient in basal dendrites and apical dendrites in CA1 in WT hippocampus. The gradient expression of Kv4.2 is impaired in the same area of AS mice. KA administration (25 mg/kg, i.p., 3 h) also reduced the Kv4.2 gradient in the same area. Line scan showed the representative Kv4.2 signal in each slice. Scale bars: 50 μm. SO: *stratum oriens*, SP: *stratum pyramidale*, SR: *stratum radiatum*, SLM: *stratum lacunosum moleculare*. **(B)** Representative images of distal /proximal SO and distal /proximal SR, and statistical analysis of Kv4.2 gradient showed in **(A)**. Data presented as Kv4.2 level in distal dendrites normalized to proximal dendrites (gradient, top and middle graph) and Kv4.2 gradient affected by KA (bottom graph). *n =* 5 mice in WT and AS group, 3 mice in WT-KA and AS-KA group. Two-three slices were used for each mouse and three-line scans for each slice. Two-way ANOVA with Fisher’s LSD test for Kv4.2 gradient in SO and SR. There are interactions between genotype and treatment for gradient in SO: *F*(1, 128) = 7.824, *p* = 0.0060, and gradient in SR: *F*(1, 128) = 10.18, *p* = 0.0018. Two-sample t-test for the Kv4.2 gradient affected by KA. *p < 0.05, **p < 0.01, ***p < 0.001. Scale bars: 5 μm.

Together with the biochemical results, these findings show that UBE3A deficiency is associated with both altered Kv4.2 complex composition and impaired spatial organization of Kv4.2 along CA1 dendrites. The reduced response to KA further suggests that UBE3A is required for the normal activity-dependent remodeling of dendritic Kv4.2 distribution.

## 3 Discussion

In the present study, we identify a trafficking-dependent mechanism by which UBE3A regulates Kv4.2 channel organization in hippocampal neurons. Our findings show that UBE3A preferentially associates with internalized, but not surface-resident, Kv4.2, indicating that UBE3A acts primarily on a post-endocytic channel pool. In parallel, we show that loss of UBE3A in AS mice reduces Kv4.2 association with its auxiliary subunit DPP6 and abolishes activity-dependent remodeling of this complex, despite preserved DPP6 expression and intact basal Kv4.2 phosphorylation. Together with the altered dendritic distribution of Kv4.2 observed in CA1 neurons, these results support a model in which UBE3A is required for normal processing of internalized Kv4.2 and for maintenance of the dendritic channel gradient that constrains excitability.

These data extend our previous model in which neuronal activity promotes p38-dependent phosphorylation of Kv4.2 at T607, recruitment of Pin1, dissociation from DPP6, and subsequent UBE3A-dependent degradation (Hu et al., 2025; Hu et al., 2020). The current results refine this sequence by placing UBE3A downstream of channel internalization. In both live-labeling and biochemical pulldown experiments, UBE3A showed minimal interaction with surface-labeled Kv4.2 when membrane trafficking was restricted, whereas the interaction increased after endocytosis was allowed (Figure 1). This suggests that UBE3A does not constitutively target Kv4.2 at the plasma membrane, but instead preferentially recognizes a post-endocytic population of the channel. Such a mechanism is consistent with a role for UBE3A in activity-coupled channel turnover rather than global control of Kv4.2 abundance (Hu et al., 2025). Consistent with this model, a separate study using surface biotinylation showed that AMPA stimulation reduced surface Kv4.2 in WT hippocampal neurons, whereas this effect was abolished in Kv4.2TA neurons (paper in press). These findings provide additional evidence that neuronal activity promotes removal of Kv4.2 from the plasma membrane and that phosphorylation/Pin1-dependent regulation is required for this process. In neurons, this would provide an approach to selectively process channels that have already undergone activity-dependent conformational or compositional changes.

In AS mice, basal phosphorylation of Kv4.2 at T602 and T607 was preserved, and PTZ-induced phosphorylation at T607 remained intact (Figure 3A). These observations indicate that upstream signaling mechanisms driving activity-dependent Kv4.2 phosphorylation are largely functional in the absence of UBE3A. Interestingly, basal Kv4.2–DPP6 association was already reduced in AS mice, and PTZ did not produce a significant additional reduction (Figure 2). One possible explanation is that increased neuronal activity associated with UBE3A deficiency has already shifted a fraction of Kv4.2 away from the DPP6-associated state, thereby partially occluding further activity-dependent dissociation. The preserved PTZ-induced T607 phosphorylation indicates that this lack of further DPP6 dissociation is unlikely to result simply from failure of the upstream phosphorylation pathway. Moreover, because DPP6 itself influences Kv4.2 phosphorylation (Figure 3B), Kv4.2 phosphorylation and DPP6 association are not necessarily expected to change in parallel. Thus, the primary defect in AS appears to occur downstream of phosphorylation, at the level of channel complex remodeling and post-endocytic processing. In this respect, the phenotype of DPP6 knockout mice is particularly informative. Unlike AS mice, DPP6-deficient mice showed abnormal Kv4.2 phosphorylation dynamics (Figure 3B), including elevated basal phosphorylation and loss of activity-dependent regulation. This suggests that DPP6 is not simply a passive auxiliary subunit but also influences the phosphorylation state of Kv4.2. Taken together, these findings support reciprocal coupling between Kv4.2 phosphorylation and DPP6 association: phosphorylation promotes complex dissociation, whereas DPP6 association helps maintain appropriate phosphorylation dynamics.

A major finding of this study is that AS mice exhibit reduced Kv4.2-DPP6 association despite normal total DPP6 expression and unchanged subcellular fractionation profiles (Figure 2). This argues against the possibility that the phenotype is due to a loss of DPP6 expression or a primary defect in DPP6 localization. Rather, the data are consistent with a model in which UBE3A deficiency leads to the accumulation of a Kv4.2 pool that has already dissociated from DPP6 and been internalized but is no longer efficiently processed. Under physiological conditions, activity-dependent dissociation of DPP6 from Kv4.2 is likely transient, because internalized channels are either degraded or recycled in a regulated manner (Ferron et al., 2021; Estadella et al., 2020). In the absence of UBE3A, however, failure of this downstream step could stabilize an intracellular, DPP6-poor Kv4.2 population. This would reduce the apparent association between Kv4.2 and DPP6 at baseline and limit further remodeling in response to stimulation. However, the present study does not directly demonstrate accumulation of this internalized Kv4.2 population in AS neurons, and this aspect of the model will require further investigation.

In addition, DPP6 has also been shown to work independently from Kv4.2 to promote filopodia growth and stability, as well as spine formation and dendritic arborization (Lin et al., 2013). DPP6 has been linked to several neurodegenerative diseases (Li et al., 2024; Liao et al., 2013; Egger et al., 2014; Naujock et al., 2020). Deletion of DPP6 causes phenotypes such as increased amyloid-β and hyper-phosphorylated tau, reduced brain volume, and deficits in learning and memory (Lin et al., 2020), which mirrors the pathology observed in patients with AD (Lin J. et al., 2022). These findings indicate that DPP6 may also serve a neuroprotective role against symptoms associated with aging and AD in addition to its involvement in neurodevelopmental processes. Therefore, decreased DPP6-Kv4.2 association in AS mice may free more DPP6 from Kv4.2 and conduct other functions that are not related to Kv4.2.

An important functional consequence of the impaired Kv4.2-DPP6 association in AS mice is the disruption of dendritic Kv4.2 organization. Both AS and DPP6 knockout mice exhibited impaired dendritic gradients of Kv4.2 in CA1 pyramidal neurons, together with reduced activity-dependent redistribution. Because the dendritic enrichment of Kv4.2 is a key determinant of back-propagating action potentials, dendritic integration, and synaptic plasticity, disruption of this gradient is likely to have broad consequences for hippocampal function. Although reduced Kv4.2–DPP6 association might appear inconsistent with our previous finding of increased A-type K^+^ current (I_A_) in AS hippocampal neurons (Hu et al., 2025), the previous I_A_ measurements were obtained from somatic outside-out patches rather than distal dendrites. DPP6 is particularly important for establishing the increasing Kv4.2-mediated current gradient along CA1 dendrites (Sun et al., 2011). Thus, increased overall Kv4.2 abundance resulting from impaired UBE3A-dependent degradation could contribute to increased somatic I_A_, while reduced Kv4.2–DPP6 association could simultaneously disrupt the normal dendritic organization of Kv4.2. Additional changes in Kv4.2 complex composition, trafficking, or channel gating may also contribute to the altered I_A_ in AS neurons. In this context, the phenotype observed in AS mice is particularly notable because the basal distribution of Kv4.2 resembled that seen in PTZ-treated wild-type animals. This suggests that loss of UBE3A may place neurons in a constitutively remodeled state that mimics seizure- or high-activity-induced channel redistribution. In fact, AS is characterized by the occurrence of spontaneous seizures (Fitzgerald, 2024; Judson et al., 2026), and AS mice on the 129Sv/Ev strain are susceptible to spontaneous seizures (Jiang et al., 1998). Neuronal hyperexcitability is considered an important component of disease pathophysiology (Wallace et al., 2012; Sun et al., 2019). Kv4.2-mediated A-type current normally dampens dendritic excitability and limits the propagation of excitatory signals (Hoffman et al., 1997). Therefore, impaired Kv4.2 trafficking and redistribution could directly promote network instability. Such a tonic shift would be expected to reduce the dynamic range of further activity-dependent regulation and could contribute to the altered excitability previously described in AS models.

From a translational perspective, these results suggest that therapeutic strategies in AS may need to address not only synaptic defects but also ion channel trafficking and spatial organization. Restoration of UBE3A function remains the most direct therapeutic approach (Samanta, 2026; Judson et al., 2026); however, our findings raise the possibility that targeting downstream components of the Kv4.2 pathway may also be beneficial. For example, interventions that stabilize Kv4.2-DPP6 complexes, normalize post-endocytic channel processing, or restore appropriate dendritic Kv4.2 distribution could potentially improve neuronal excitability control. Whether such approaches would reduce seizure susceptibility or improve cognitive function in AS will require further investigation.

In conclusion, our data supports a model in which neuronal activity triggers Kv4.2 phosphorylation and dissociation from DPP6, followed by channel internalization and UBE3A-dependent processing of the post-endocytic channel pool. In wild-type neurons, this pathway enables dynamic turnover and redistribution of Kv4.2, thereby maintaining the dendritic gradient required for proper control of excitability. In AS, loss of UBE3A uncouples internalization from downstream processing, leading to accumulation of a remodeled, DPP6-poor Kv4.2 population, impaired dendritic redistribution, and a basal state resembling seizure-induced channel remodeling. These findings identify UBE3A as a key regulator of activity-dependent Kv4.2 homeostasis and provide a mechanistic framework linking UBE3A deficiency to dendritic dysfunction and hyperexcitability in Angelman syndrome.

## 4 Materials and methods

### 4.1 Expression constructs

The human constructs were purchased from Origene: Myc-DDK-DPP6 (RC216919), Myc-DDK-UBE3A (RC200629). The rat DPP6, KChIP2, and BBS-Kv4.2 constructs were generated and used previously (Tabor et al., 2019).

### 4.2 Chemicals

Chemicals were purchased: Kainic acid (KA, Sigma, K0250), pentylenetetrazole (PTZ, Sigma, P6500). For intraperitoneal (IP) injections, KA and PTZ were dissolved in saline.

### 4.3 Antibodies

Primary antibodies: Mouse anti-Kv4.2 (NeuroMab, 75–016) was used at 1:2000 for western blot, 1:200 for immunostaining; rabbit anti-Kv4.2 (Sigma, HPA029068) was used at 1:2000 for western blot, 1:400 for staining; Kv4.2-pT602 (Santa Cruz, SC-16983-R) was used at 1:1000 for western blot; Kv4.2-pT607 (Santa Cruz, SC-22254-R) was used at 1:500 for western blot; DPP6 (SB biological, 100592-T36) was used at 1:2000 for western blot; UBE3A (Sigma, SAB1404508) was used at 1:2000 for western blot, 1:200 for immunostaining; PSD95 (Invitrogen, MA1-045) was used at 1:2000 for western blot; Myc (Millipore, 05–419) was used at 1:10000 for western blot; Flag (Sigma, F3165) was used at 1:10000 for western blot; Actin (Sigma, A-1978) was used at 1:10000 for western blot.

Secondary antibodies: Alexa Fluor 488 goat anti-mouse (Invitrogen, A-11029) was used at 1:500; Alexa Fluor 488 goat anti-rabbit (Invitrogen, A-11034) was used at 1:500; Alexa Fluor 555 goat anti-mouse (Invitrogen, A-21424) was used at 1:500; Alexa Fluor 555 goat anti-rabbit (Invitrogen, A-21429) was used at 1:500; IRDye 680 goat anti-mouse (Invitrogen, A-21057) was used at 1:10000, IRDye 680 goat anti-rabbit (Invitrogen, A-21076) was used at 1:10000. IRDye 800CW goat anti-mouse (Licor, 926–32210) was used at 1:10000, IRDye 800CW goat anti-rabbit (Licor, 926–32211) was used at 1:10000.

### 4.4 Mouse models

AS mice were obtained from Dr. Ben Philpot’s lab (UNC-Chapel Hill). AS mice and WT littermates were obtained by crossing a paternal-null heterozygous female (*Ube3a^m+/p-^* mice) with a C57BL/6 J male, resulting in either WT progeny or progeny with loss of UBE3A only on the maternally inherited allele (*Ube3a^m−/p+^* or AS mice). Adult male mice were used in this study.

Mice were group-housed in plastic mouse cages with free access to standard rodent chow and water. The colony room was maintained at 22 ± 2 °C with a 12 h: 12 h light: dark cycle. All mice were housed and bred in the Porter Neuroscience Research Center animal facility at the National Institutes of Health in Bethesda, MD. All animal protocols and procedures were performed in accordance with guidelines approved by the *Eunice Kennedy Shriver* National Institute of Child Health and Human Development Animal Care and Use Committee and in accordance with NIH guidelines.

### 4.5 Cell culture and transfection

HEK293T cells used in biochemistry and immunostaining experiments were obtained from Dr. Paul Worley’s lab (Hu et al., 2012). HEK293T cells were cultured in DMEM medium containing 10% FBS. Transfections were performed with X-tremeGENE 9 according to the manufacturer’s specifications. Cells were harvested approximately 40 h after transfection.

### 4.6 Bungarotoxin (BTX) labeling of HEK293T cells

Living cells transfected with BBS-tagged Kv4.2 and UBE3A constructs were cultured at 37 °C for approximately 40 h. Cells were cooled to 17 °C before Rh-BTX labeling to minimize trafficking, and then labeled with 2 μg/mL rhodamine-conjugated bungarotoxin (Rh-BTX, Molecular Probes, T1175) in culture medium at 17 °C for 30 min. The cells were then washed to eliminate free Rh-BTX and incubated at 37 °C for 50 min. Control cells were incubated at 4 °C after BTX-labeling to limit trafficking. Cells were fixed with 4% PFA and permeabilized with 0.2% Triton X-100 in PBS. Cells were then incubated with mouse anti-Ube3A antibody for 2 h at RT. After washing, cells were incubated with anti-mouse-488 secondary antibody at RT for 1 h. Cells were then mounted on slides with anti-fade mounting medium containing 4′,6-diamidino-2-phenylindole (DAPI, Invitrogen, P36962) and imaged using a Zeiss 710 laser scanning confocal microscope equipped with a 63x objective. The images were collected using ZEN analysis software (Zeiss) and analyzed with ImageJ (NIH) analysis software.

For the BTX-Biotin pulldown experiment, HEK293T cells (3.5 × 10^5^ cells per well) were plated into Corning 6-well dishes that had been coated with 1 mL of 0.5 mg/mL poly-L-ornithine (Sigma, P3655) in 0.1 M Trizma pH 8.5 (Sigma T1194) for approximately 4 h and then washed three times with 2 mL PBS immediately before use. Cells transfected with BBS-tagged Kv4.2 and Ube3A constructs were labeled with 2 μg/mL biotin-conjugated bungarotoxin (BTX-Biotin, Molecular Probes, T1196) in culture medium at 17 °C for 30 min. The cells were then washed to eliminate free BTX-Biotin and incubated at 37 °C for 20- or 50- min. Control cells were incubated at 4 °C for 50 min after BTX-labeling to limit trafficking. Cells were lysed with lysis buffer (1 X PBS, pH 7.4, with 1% Triton X–100, phosSTOP and Complete™ EDTA–Free protease inhibitors) and sonicated. After centrifugation, the supernatant was mixed with 25 μL Streptavidin beads for 2 h at 4 °C. After washing three times with lysis buffer, the samples were eluted with SDS loading buffer and analyzed by gel electrophoresis and western blotting.

### 4.7 Subcellular fractionation

Subcellular fractionation was described previously (Pacchioni et al., 2009). Briefly, Mouse hippocampi were harvested and homogenized in 0.5 mL ice cold 0.32 M sucrose buffer (0.32 M sucrose in 10 mM HEPES buffer, pH 7.4 with proteinase inhibitors) about 18–20 stokes on ice. Samples were centrifuged at 1,000 g for 10 min at 4 °C. The supernatant was transferred to a new tube and centrifuged at 12,000 g for 15 min at 4 °C. The supernatant was removed and the pellet was resuspended in 200 μL of 4 mM HEPES buffer (4 mM HEPES, 1 mM EDTA, pH 7.4 with proteinase inhibitors) and centrifuged at 12,000 g for 15 min at 4 °C. The supernatant was removed and the pellet wash was repeated once. The pellet was then resuspended with 150 μL Buffer A (20 mM HEPES, 100 mM NaCl, 0.5% Triton-X, pH 7.2 with proteinase inhibitors) and rotated slowly at 4 °C for 15 min. 50 μL samples were saved as a synaptoneurosome fraction and then the remaining was centrifuged at 12,000 g for 15 min at 4 °C. The supernatant was then saved as the non-PSD fraction, and the pellet as the PSD fraction. All the samples were then mixed with sample loading buffer for western blot assay.

### 4.8 Co–immunoprecipitation and immunoprecipitation assays

HEK293T cells were used in co–immunoprecipitation assays as previous reported (Hu et al., 2012). For the DPP6-UBE3A co-IP experiment, co-IP buffer (1 X PBS, pH 7.4, with 1% Triton X–100, phosSTOP and Complete™ EDTA–Free protease inhibitors) were added (400 μL for a 6-well of HEK293T cells), and the samples were sonicated. After centrifugation, the supernatant was mixed with 2 μg of UBE3A antibody for 3–4 h at 4 °C. Next, 25 μL of protein G magnetic beads (Bio-Rad, 161-4,023) were added for an additional 2 h or overnight. The protein beads were washed three times with co-IP buffer. The protein samples were eluted with SDS loading buffer and analyzed by gel electrophoresis and western blotting. For the Kv4.2-DPP6 co-IP experiment, co-IP buffer (1 X PBS, pH 7.4, with 1% Triton X–100, 0.2% Chaps, phosSTOP and Complete™ EDTA–Free protease inhibitors) were added (1:20 w/v for brain tissue), and the samples were sonicated. After centrifugation, the supernatant was mixed with 3–4 μg of Kv4.2 antibody (NeuroMab, 75-016) for 3 h at 4 °C. Next, 50 μL of 1:1 protein G–Sepharose slurry (GE Healthcare, 17-0886-02) was added for an additional 3 h. The protein beads were washed three times with IP buffer. The protein samples were eluted with SDS loading buffer and analyzed by gel electrophoresis and western blotting.

### 4.9 Western blot and quantification

Protein samples were mixed with 4x LDS sample buffer (Invitrogen NP0007) and 10x sample reducing agent (Invitrogen NP0009) to a final concentration of 1x. Samples were loaded on 4%–12% Bis-Tris gradient gel (Invitrogen 12-well, NP0322; 15-well, NP0323). The proteins were transferred to Immobilon-FL PVDF membrane (EMD Millipore, IPFL00010). The membrane was blocked with Odyssey blocking buffer (Li-COR, 927-40,000) for 1 h at room temperature, followed by incubation with primary antibody in PBS overnight at 4 °C. The membrane was then washed with PBST (PBS, pH 7.4, and 0.1% Tween-20) three times and incubated with secondary antibody in PBS for another hour. After three washes with PBS, the membrane was scanned using an Odyssey imaging system (LI-COR) according to the manufacturer’s protocol. Quantification of western blots was carried out using the gel analysis function in ImageJ within the linear range of detection which is determined by using serial dilutions of a representative sample.

### 4.10 Immunostaining and quantification

Brain slices were washed with PBS and then permeabilized and blocked with 0.25% Triton X−100 + 3% horse serum in PBS at RT for 2 h. Brain slices were incubated with mouse anti-Ube3A antibody and rabbit Kv4.2 antibody at 4 °C overnight. After washing, slices were incubated with anti-mouse-488 and anti-rabbit-555 secondary antibodies at RT for 2 h. After washing, slices were then mounted on slides with anti-fade mounting medium containing 4′,6-diamidino-2-phenylindole (DAPI, Invitrogen, P36962) and imaged using a Zeiss 710 laser scanning confocal microscope equipped with a 63 × and 20 × objectives.

Images were quantified by ImageJ. For Kv4.2 gradient distribution, line scan was utilized to showed staining signals from *stratum oriens* to *stratum lacunosum*. The averaged intensities of 10 μm dendrites in proximal (about 10 μm away from stratum pyramidale) and distal (about 70 μm for stratum oriens and 130 μm for stratum radiatum away from stratum pyramidale) areas were calculated.

### 4.11 Statistical analysis

Biochemistry and behavioral data were analyzed by Origin 2019b with two-sample t-test and GraphPad Prism 9.5 with One-Way ANOVA and Two-Way ANOVA where appropriate. Results were considered significant when *p <* 0.05. All the data are presented as mean ± SEM.

## Data Availability

Original data sets have been deposited online and made publicly available (Mendeley Data, V1, doi: 10.17632/32r95yd8rk.1).
